# Supplementary material for: Spatial Modelling of Soil-Transmitted Helminth Infections in Kenya: A Disease Control Planning Tool
Source: PLoS Negl Trop Dis. 2011 Feb 8;5(2):e958. doi: 10.1371/journal.pntd.0000958 (PMC3035671; doi:10.1371/journal.pntd.0000958)
Supplement: Text S1 — Model description. (0.25 MB RTF) [file pntd.0000958.s001.rtf]

Supporting Text S1 – Model Description

The number of examined (ni,j,t) and egg-positive (Yi,j,t) individuals for each species in survey i (i=1,…,N) at location j (j = 1,….,J) in year t (tj=1,…,T) were modelled as binomial variates in the form:


	 
where á is the intercept,   the matrix of survey covariates,   the matrix of environmental covariates,  a geostatistical random effect modelled using an isotropic, stationary exponential decay function [28]:  where  is the straight-line distance between pairs of points a  and b, and  is the rate of decline of spatial correlation, and  a temporal random effect modelled using a first-order conditional autoregressive (CAR) prior structure[1], where a simple adjacency matrix was specified with a weight of one given to consecutive years and a weight of zero given to non-consecutive years.  Non-informative priors were used for  and the coefficients (normal prior with mean 0 and precision 1 x 10^6^), the prior distribution of  was uniform with upper and lower bounds set at 0.05 and 50 and the precision of  and  were given non-informative gamma distributions. 

Model fit was carried out in WinBUGs 1.4.1 (Imperial College London, and MRC, London, UK).  Following a burn-in of 9,000 iterations, the values for the intercept and coefficients were stored for 1,000 iterations and model convergence was assessed using diagnostic tests for convergence and by visually inspecting the time series plots. Convergence was successfully achieved after 10,000, and the model was run for a further 5,000 iterations with thinning every five iterations, during which prediction locations were stored for 2009 and 1989.  Model output therefore consisted of samples from the posterior distribution mean STH prevalence at each grid location for 2009 and 1989, which were used to generate point estimates (the mean of each set of posterior samples; fig 4a-b of the main text) and endemicity class membership probabilities (the proportion of posterior samples for each grid location falling in the various class ranges). These latter values were used to present probability contour maps for the endemicity class >20% prevalence (Fig 4c-d in the main text) and intervention districts based on endemicity (Fig 5 in the main text). 


1. Besag J, York J, Mollie A (1991) Bayesian image restoration, with two applications in spatial statistics. Ann Instit Stat Maths 43: 1-59.
