# Supplementary material for: Spatial Modelling of Soil-Transmitted Helminth Infections in Kenya: A Disease Control Planning Tool
Source: PLoS Negl Trop Dis. 2011 Feb 8;5(2):e958. doi: 10.1371/journal.pntd.0000958 (PMC3035671; doi:10.1371/journal.pntd.0000958)
Supplement: Text S2 — Additional results to support those provided in the main article. (95.82 MB RTF) [file pntd.0000958.s002.rtf]

Supporting Text S2 – Additional Results


Table S1  Estimates of univariable non-spatial logistic regression models of soil-transmitted helminth infections in Kenya (1974-2009)
	

Variable		Posterior mean (95% CI)	
		A. lumbricoides	Hookworm	T. trichiura	
	Number surveys (%) 	OR (95% CI)
p	OR (95% CI)
p	OR (95% CI)
p	
Community based surveys 
(vs. school based)	27 (2.9%)	0.59  (0.32, 1.09)
0.09	0.71  (0.45, 1.12)
0.1	0.37  (0.23, 0.57)
0.09	
Other diagnostic method 
(vs. Kato Katz)	388 (41.1%)	2.21  (1.78, 2.73)
<0.001	0.38  (0.31, 0.45)
<0.001	0.26  (0.21, 0.24)
<0.001	
max LST * (mean (range))	0 (-4.61, 3.09)	0.64  (0.58, 0.70)
<0.001	1.19  (1.04, 1.28)
<0.001	1.32  (1.21, 1.44)
<0.001	
Elevation *(mean (range))	0 (-1.75, 2.96)	1.78  (1.58, 2.01)
<0.001	0.72  (0.66, 0.79)
<0.001	0.59  (0.53, 0.66)
<0.001	
Precipitation *(mean (range))	0 (-2.94, 2.51)	1.81  (1.63, 2.01)
<0.001	1.33  (1.22, 1.44)
<0.001	1.22  (1.11, 1.34)
<0.001	
EVI (mean (range))	0.35 (0, 0.52)	2.52  (2.09, 3.05)
<0.001	1.71  (1.50, 1.95)
<0.001	2.22  (2.01, 18.6)
<0.001	
Distance to permanent water bodies (mean (range))	0.07 (0, 0.93)	6.09  (2.30, 16.15)
<0.001	0.02  (0.01, 0.06)
<0.001	6.42  (1.39, 29.6)
<0.001	

OR, odds ratio; 95% CI, 95% confidence interval; p, wald test p value; EVI, enhanced vegetation index; max LST, maximum land surface temperature; distance to permanent water bodies expressed in decimal degrees.
* Variables were standardised to have a mean of 0 and a standard deviation of 1. 	


Table S2  Bayesian hierarchical logistic regression models of A. lumbricoides in Kenya (1974-2009), comparing the full data set with a selective dataset	

Variable	Posterior mean OR (95% CI)	
	Full data set	Kato katz only	
Community based (vs. school based)	0.72	(0.51,0.96)	0.58	(0.32, 0.95)	
Other diagnostic method (vs. KK)	0.90	(0.72,1.1)			
max LST *	1.18	(0.93,1.46)	1.07	(0.87, 1.29)	
Elevation *	1.32	(0.84,1.80)	1.12	(0.75, 1.40)	
Precipitation *	1.01	(0.87,1.15)	0.93	(0.76, 1.17)	
EVI	2.57	(0.75,5.86)	5.20	(0.58, 17.31)	
Distance to permanent water bodies	0.77	(0.26,1.92)	1.22	(0.70, 5.55)	
					
 (rate of decay of spatial correlation)	6.83	(4.94, 9.02)	8.56	(5.81, 11.58)	
2space (variance of spatial random effect)	4.03	(3.05,3.97)	2.50	(1.90, 3.43 )	
2time (variance of temporal random effect)	1.22	(0.56, 2.45)	2.53	(1.09, 5.24)	

CI, Bayesian credible interval; EVI, enhanced vegetation index; max LST, maximum land surface temperature.
* Variables were standardised to have a mean of 0 and a standard deviation of 1. 	


Table S3 Bayesian hierarchical logistic regression models of hookworm in Kenya (1974-2009), comparing the full data set with a selective dataset	

Variable	Posterior mean OR (95% CI)	
	Full data set	Kato katz only	
Community based (vs. school based)	0.85	(0.57, 1.21)	0.94	(0.63, 1.36 )	
Other diagnostic method (vs. KK)	0.68	(0.53, 0.84)			
max LST *	0.84	(0.73, 0.96)	0.70	(0.54, 0.88)	
Elevation *	0.74	(0.60, 0.95)	0.62	(0.47, 0.88)	
Precipitation *	1.13	(0.98, 1.28)	1.18	(0.99, 1.41)	
EVI	0.62	(0.11, 1.64)	0.51	(0.06, 1.53)	
Distance to permanent water bodies	1.24	(0.33, 3.14)	0.48	(0.11, 1.45)	
					
 (rate of decay of spatial correlation)	8.62	(6.35, 10.93)	12.03	(7.89, 18.12)	
2space (variance of spatial random effect)	3.53	(2.83,4.53)	1.82	(1.38, 2.45)	
2time (variance of temporal random effect)	0.66	(0.32, 1.36)	0.26	(0.10, 0.59)	

CI, Bayesian credible interval; EVI, enhanced vegetation index; max LST, maximum land surface temperature.
* Variables were standardised to have a mean of 0 and a standard deviation of 1. 	


Table S4  Bayesian hierarchical logistic regression models of T. trichiura in Kenya (1974-2009), comparing the full data set with a selective dataset 	

Variable	Posterior mean OR (95% CI)	
	Full data set	Kato katz only	
Community based (vs. school based)	0.45	(0.29, 0.68)	0.53	(0.29, 0.90)	
Other diagnostic method (vs. KK)	0.43	(0.31, 0.57)			
max LST *	0.83	(0.73, 0.99)	0.98	(0.77, 1.23)	
Elevation *	0.75	(0.61, 0.93)	0.98	(0.68, 1.35)	
Precipitation *	0.91	(0.77, 1.04)	0.87	(0.71, 1.10)	
EVI	3.58	(0.47, 8.16)	2.34	(0.17, 11.2)	
Distance to permanent water bodies	1.20	(0.34, 3.19)	0.62	(0.06, 2.19)	
					
 (rate of decay of spatial correlation)	7.06	(5.15, 9.15)	30.1	(12.6, 65.78)	
2space (variance of spatial random effect)	3.55	(2.78, 4.63)	1.73	(1.33, 2.32)	
2time (variance of temporal random effect)	0.87	(0.39, 1.67)	1.05	(0.40, 0.95	

CI, Bayesian credible interval; EVI, enhanced vegetation index; max LST, maximum land surface temperature.
* Variables were standardised to have a mean of 0 and a standard deviation of 1. 	


Figure S1: Time series of temporal random effects for (A) A. lumbricoides (B) hookworm and (C) T. trichiura infection prevalence for Kenya from 1974-2009, comparing models run on the full dataset with selective datasets. Values are on a log scale (values of <0 indicate lower than average odds, values >0 indicate higher than average odds). 

(A)

(B)


(C)


Figure S2 Continuous predicted prevalence and probability contour maps for hookworm in Kenya. Probability contour maps show the probability that prevalence exceeds 20%.  Estimates of predicted prevalence are the mean posterior predictive values from a Bayesian space-time model for (a) 2009 and (b) 1989. The probability contour maps show the spatial distribution of probability that prevalence is > 20% for (c) 2009 and (d) 1989. Biological transmission limits were estimated using maximum LST (land surface temperature), assuming no transmission when maximum LST exceeds 40oC.


Figure S3 Continuous predicted prevalence and probability contour maps for Ascaris lumbricoides in Kenya. Probability contour maps show the probability that prevalence exceeds 20%.  Estimates of predicted prevalence are the mean posterior predictive values from a Bayesian space-time model for (a) 2009 and (b) 1989. The probability contour maps show the spatial distribution of probability that prevalence is > 20% for (c) 2009 and (d) 1989. Biological transmission limits were estimated using maximum LST (land surface temperature), assuming no transmission when maximum LST exceeds 40oC.


Figure S4 Continuous predicted prevalence and probability contour maps for Trichuris trichiura in Kenya. Probability contour maps show the probability that prevalence exceeds 20%.  Estimates of predicted prevalence are the mean posterior predictive values from a Bayesian space-time model for (a) 2009 and (b) 1989. The probability contour maps show the spatial distribution of probability that prevalence is > 20% for (c) 2009 and (d) 1989. Biological transmission limits were estimated using maximum LST (land surface temperature), assuming no transmission when maximum LST exceeds 40oC.
